# Adiponectin Levels Are Reduced While Markers of Systemic Inflammation and Aortic Remodelling Are Increased in Intrauterine Growth Restricted Mother-Child Couple

**DOI:** 10.1155/2014/401595

**Published:** 2014-06-22

**Authors:** Silvia Visentin, Annunziata Lapolla, Ambrogio Pietro Londero, Chiara Cosma, Mariagrazia Dalfrà, Martina Camerin, Diego Faggian, Mario Plebani, Erich Cosmi

**Affiliations:** ^1^Department of Woman's and Child's Health, Maternal Fetal Medicine Unit, University of Padua School of Medicine, Padua, Italy; ^2^Medical and Surgical Science, University of Padua, Padua, Italy; ^3^Clinic of Obstetrics and Gynecology, Department of Experimental Clinical and Medical Science, DISM, University of Udine, Udine, Italy; ^4^Laboratory Medicine, University of Padua, Padua, Italy; ^5^Maternal and Fetal Medicine Unit, Obstetrics and Gynecology, University of Padua School of Medicine, Via Giustiniani No. 3, 35128 Padua, Italy

## Abstract

*Aim of the Study*. To investigate the relationships between the adipocytokine levels, markers of inflammation, and vascular remodelling in pregnancies complicated by intrauterine growth restriction (IUGR). *Materials and Methods*. This was a retrospective study. One hundred and forty pregnant patients were enrolled. Adiponectin, leptin, tumor necrosis factor *α* (TNF*α*), interleukin-6 (IL-6), and C reactive protein (CRP) were assessed in IUGR, small for gestational age (SGA), and appropriate for gestational age (AGA) mother-child couples at delivery. IUGR and SGA fetuses were defined as fetuses whose estimated fetal weight (EFW) was below 10th percentile for gestational age with and without umbilical artery (UA) Doppler abnormalities, respectively. Fetal aorta intima media thickness (aIMT) was evaluated by ultrasound in the same fetal groups. Data were analyzed by R (version 2.15.2). *Results*. There were 37 IUGR mother-child couples, 33 SGA, and 70 AGA. Leptin, TNF*α*, IL-6, and CRP serum levels were higher in IUGR pregnant patients (*P* < 0.05). Adiponectin levels were significantly reduced in IUGR fetuses compared to SGA and AGA, while leptin, TNF*α*, and IL-6 levels were higher in IUGR group (*P* ≤ 0.05). Fetal aIMT was significantly higher in IUGR (*P* < 0.05) and in this group there was a negative correlation between aIMT and adiponectin/leptin ratio (A/L ratio) (*P* < 0.05) and between adiponectin and IL-6 levels (*P* < 0.05). *Conclusions*. In conclusion, compared to SGA and AGA, IUGR fetuses had reduced circulating levels of adiponectin and elevated measures of aIMT and several inflammatory markers. Moreover, adiponectin levels were negatively correlated with aIMT in IUGR fetuses suggesting a possible causal link between reduced adiponectin and vessel remodelling.

## 1. Introduction

Intrauterine growth restriction (IUGR) is considered the second leading cause of perinatal morbidity and mortality [1]. Adverse perinatal environments influence fetal growth and may result in developmental adaptations that permanently change the physiology and metabolism of the offspring thereby predisposing individuals to metabolic, endocrine, and cardiovascular events [2]. Insulin resistance has been proposed to be the underlying pathogenic link between metabolic syndrome and cardiovascular disease [3]; both are associated with a state of low-grade aseptic markers of systemic inflammation, whose pathogenic significance was mostly eclipsed by the vigorous advances in lipid research [4]. A growing body of evidence has recently suggested that the adipose tissue may play a major role in linking poor fetal growth to subsequent development of adult diseases [5]. IUGR is known to alter the development of fetal adipose tissue. An increase in sympathetic tone and a dyslipidemic condition (high concentration of apolipoprotein B and apolipoprotein A1 and reduction in the concentration of insulin-like growth factor 1) in IUGR fetuses could help to increase the existing endothelial damage [6]. Several human studies showed that an estimated fetal weight (EFW) below the 10th centile and fetal Doppler vessel abnormalities were associated in utero and in neonates with endothelial dysfunction, represented by a higher aorta intima media thickness (aIMT) [7, 8]. In recent years adipocyte-derived signaling molecules (“adipokines”) have been implicated in intrauterine growth restriction disorders. Adipose tissue is a complex organ including adipocytes, immune cells, fibroblast, tissue resident macrophages, collagen fibers, and vascular constituents. Over the past decade, it has been recognized that it is not only a fat store tissue, but also an endocrine organ, secreting a variety of bioactive molecules which influence body metabolism and energy homeostasis [9]. High serum concentrations of proinflammatory cytokines, such as leptin, CRP, IL-6 and TNF-*α*, as well as a reduction in serum adiponectin levels, should be related to low intrauterine weight and might worsen this condition [10].

Circulating levels of TNF*α* and IL-6 are directly correlated with adiposity and insulin resistance [11]. Macrophages, crucial contributors to inflammation, are the major source of TNF*α* produced by white adipose tissue (WAT) and contribute approximately 50% to WAT-derived IL-6 [12]. CRP is released by adipose tissue and is an important first line host defense molecule; it recognizes damaged cells and promotes their elimination by activating the complement system [13]. Plasma leptin concentrations directly reflect the amount of adipose tissue and the control of appetite is its primary role [14]. Leptin action in regulating immunity has been fueled by early observations in animal models, protecting T lymphocytes from apoptosis and regulating T-cell activation [15]. Leptin also influences monocytes activation, phagocytosis, and cytokine production; in endothelial cells it finally induces oxidative stress and upregulation of adhesion molecules [15]. Adiponectin is the most abundant adipokine produced by the adipose tissue and belongs to a collagen superfamily, sharing significant homology with collagen X, VIII, complement factor C1q, and TNF-*α*, suggesting a connection with the immune system. It modulates insulin action and exerts anti-inflammatory effects, playing an important role in the pathogenesis of metabolic syndrome [16]. Several reports suggest that adiponectin exerts an antiatherogenic role protecting vessels from endothelial dysfunction by its quiescent effect on macrophages, suppressing their production of proinflammatory cytokines, such as TNF*α* and IL-6, and inducing the production of anti-inflammatory cytokines [16, 17]. The mechanism underlying the relationship between birth weight, inflammation, and insulin sensitivity during adulthood remains still unclear.

To investigate the hypothesis that fetuses with a low EFW and umbilical artery (UA) Doppler abnormalities would exhibit lower concentrations of serum adiponectin and higher levels of leptin, CRP, and proinflammatory cytokines (TNF*α* and IL-6), we studied these adipocytokines in IUGR mother-child couples in comparison with small for gestational age (SGA) and appropriate for gestational age (AGA).

## 2. Subjects and Methods

### 2.1. Population

A retrospective study was performed from January, 2011, to March, 2013, in the Department of Woman and Child Health, University of Padua, Italy. The protocol was designed to study fetuses that were IUGR and SGA and those that were AGA. They were selected during the ultrasound evaluation of the third trimester. One hundred and forty pregnant patients were included in the study. IUGR fetuses were classified as fetuses whose EFW was below the 10th percentile for gestation age with UA Pulsatility Index (PI) > 2 SD; SGA fetuses were those whose EFW was below the 10th percentile without fetal velocimetry abnormalities. All pregnancies were dated correctly by first trimester ultrasound scan until the twentieth week of gestation. Customized centile were used with respect to the Italian standards of referral [18]. The Ethical Committee of the University Hospital approved the study protocol and all included mothers provided signed informed consent before enrollment. The diagnosis of IUGR and SGA was made within the 32nd week of gestation. Excluding factors were twin pregnancy, major congenital anomalies, pregnancies complicated by maternal history of cardiovascular disease or endocrine disorders (diabetes, hypercholesterolemia, preeclampsia, thyroid, and adrenal problems), and clinical chorioamnionitis. Women who consumed alcohol, smoked, nicotine, or any medication such as ritodrine and corticosteroids (except for fetal lung maturation) were excluded, such as amniotic fluid disorders and placental abnormalities. Antenatal surveillance was performed by fetal biometry every two weeks and maternal-fetal Doppler and amniotic fluid evaluation from one up to three times a week, depending on severity. Mean PI values were found to be upper 95th centile in all IUGR fetuses with a progressive worsening in 11 ones (UA absent end diastolic flow, PI middle cerebral artery (MCA) < 2 SD, a reduction of a wave in ductus venosus), indicating the initiation of fetal blood flow redistribution.

Amniotic fluid, as assessed by the largest fluid column on the vertical plane, was decreased (<2 cm) in the same 11 IUGR fetuses. PI uterine arteries were altered in 25 IUGR fetuses. In the AGA group, mother were healthy and no smokers.

aIMT and diameter measurements were determined for each fetus at a mean gestational age of 32 weeks (range 30 to 34 weeks). All parameters were measured by high-resolution ultrasound scan using an ultrasound machine equipped with a 3.5- to 5-MHz linear array transducer (Antares, Siemens Medical Solutions, Mountain View, CA). aIMT and diameter were measured in a coronal or sagittal view of the fetus at the dorsal arterial wall of the most distal 15 mm of the abdominal aorta sampled below the renal arteries and above the iliac arteries; gain settings were used to optimize image quality. Abdominal aIMT was defined as the distance between the leading edge of the blood-intima interface and the leading edge of the media-adventitia interface on the far wall of the vessel, as previously described [8, 19]. Three measurements were taken, and the arithmetic mean aIMT was considered for the study. All images were taken at end-diastole of the cardiac cycle to minimize the variability. All the ultrasound studies in fetuses and children were performed by two, independently, blinded, skilled practitioners (E.C, S.V.). Before starting the main research, the intraobserver and interobserver agreement were evaluated in the measurement of aorta intima media thickness (0.876 and 0.856, resp.). Data concerning women, pregnancies, and deliveries were recorded according to the routine practice of the Department of Obstetrics and Gynecology of the University of Padua. During gestation mother's age, BMI before and after gestation, parity, and obstetrical history were collected. At delivery, sex, gestational age, birth weight, length, mode of delivery, Apgar score, acid base equilibrium, and perinatal data were registered. The main clinical features are reported in Table 1.

### 2.2. Collection of Blood Sample

Maternal blood was collected during the first stage of labor or before receiving anesthesia in case of elective cesarean section. Umbilical vein samples were collected from doubly clamped umbilical cords, after fetal expulsion, from all IUGR, SGA, and controls. Serum leptin, adiponectin, TNF*α*, IL-6, and CRP were measured.

Blood was put in sterile, pyrogen-free tubes and it was centrifuged (3000 g/min for 10 min at 5°C) after clotting; the supernatant serum was kept frozen at −80°C until assay.

### 2.3. Maternal and Cord Serum Assays

Serum leptin levels were measured using the Kit Leptin (Mediagnost, CAT. R44, Germany), a radioimmunoassay with streptavidin coated tubes. Analytical specifications are analytical sensitivity = 0.1 *μ*g/L; intra-assay and interassay variation (CV%), respectively, 4.4 and 5.1; measuring range = 0.1–64 *μ*g/L.

Serum adiponectin levels were measured using the radioimmunoassay RIA KIT Human Adiponectin (Millipore, cat. number HADP-61HK). Analytical specifications are limit of sensitivity = 1 *μ*g/L; measuring range = 1–240 *μ*g/L; intra-assay and interassay imprecision (CV%), respectively, 3.59 and 7.85.

Human TNF*α* was measured using the analyzer IMMULITE One (Medical System S.p.A., Genova, Italia). Assay characteristics are measuring range = 1,7–1000,0 ng/L; analytical sensitivity = 1,7 ng/L; between assays imprecision = 17,0–788,0 ng/L (CV = 4,0–6,5%). Each sample was measured in triplicate and each experiment was repeated three times.

Serum IL-6 was measured using the analyzer IMMULITE One (Medical System S.p.A., Genova, Italia). The test is an immunoassay based on chemiluminescence. Assays characteristics are measuring range = 2,0–1000,0 ng/L; analytical sensitivity = 2,0 ng/L; between assays imprecision = 88,0–1001,0 ng/L (CV = 5,1–7,5%).

Serum CRP level determination was performed by fully mechanized latex-particle-enhanced immunonephelometric assays on the Dimension Vista (Siemens Healthcare Diagnostic Products GmbH). Intra- and interassay coefficients of variation were 11.91 mg/L, 4.8%, and 6.0%.

### 2.4. Statistical Analysis

Statistical analysis was performed using R system. The normal distribution of the data was determined using the Kolmogorov-Smirnov test. These data were analyzed using, when appropriate, the following tests: *t*-test, Wilcoxon test, chi-square test, or Fisher's exact test. Kendall's Tau was used for correlation analysis. All possible correlations were performed and only significant correlations are reported in the text. A *P* value < 0.05 was accepted as statistically significant.

## 3. Results

### 3.1. Description of the Groups

There were 37 IUGR, 33 SGA, and 70 AGA mother-child couples. Table 1 shows the characteristics of the samples. We found no significant differences in maternal age or parity among studied groups (mean age 32.30 ± 4.88). Gestational age at delivery and neonatal weight were significantly lower in IUGR pregnancies than in other groups (*P* < 0.05). aIMT was higher in IUGR fetuses than in SGA and AGA (*P* < 0.05), and SGA fetuses had a higher aIMT than controls (*P* < 0.05).

### 3.2. Maternal and Fetal Hormones Levels

#### 3.2.1. Adiponectin

Within each group adiponectin levels in the mother-fetus couples were higher in the fetuses (*P* < 0.05). There was a statistically significant lower level of maternal adiponectin concentration in IUGR than in control group (*P* < 0.05). Also in IUGR fetuses' adiponectin levels were lower than in AGA and SGA groups (*P* < 0.05). No differences were observed between SGA and AGA fetuses (Table 2).

#### 3.2.2. Leptin

IUGR and SGA women presented higher leptin serum concentrations than AGA (*P* < 0.05). IUGR fetuses presented higher leptin levels than SGA and AGA (*P* < 0.05). There were no differences in leptin fetus levels between SGA and AGA (Table 2).

#### 3.2.3. IL-6, TNF*α* and CRP

IL-6 concentration was higher in IUGR fetuses than SGA and AGA (*P* < 0.05); there was also a significant difference between SGA and control group (*P* < 0.05) (Table 2).

TNF*α* levels in mother-fetus couples were significantly higher in IUGR than SGA and controls. (*P* < 0.05).

IUGR patients presented maternal serum CRP concentrations higher than SGA and AGA (*P* < 0.05). Fetal IUGR, SGA, and AGA serum CRP did not show differences (Table 2).

### 3.3. Correlation among Maternal-Fetal Hormone Levels, Anthropometric, and Ultrasound Measures

**Figure 1:**
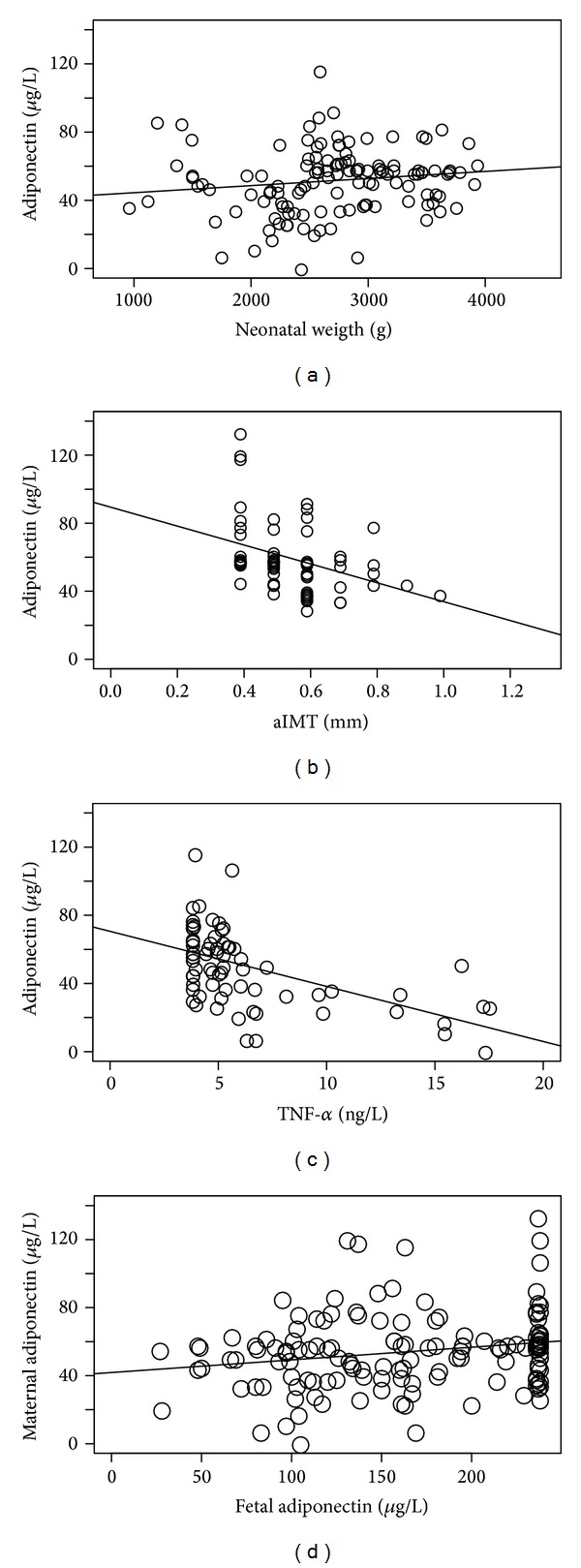
Correlations of maternal blood adiponectin level with other hormone levels, anthropometric, or ultrasound parameters. (a) Correlation between neonatal weight and maternal blood adiponectin levels in all the population (tau test *P* < 0.05). (b) Correlation between fetal aIMT and maternal blood adiponectin levels in AGA population (*P* < 0.05). (c) Correlation between maternal blood TNF*α* and adiponectin levels in IUGR and SGA the population (*P* < 0.05). (d) Correlation between neonatal and maternal blood adiponectin levels in all the population (*P* < 0.05).

In all groups, maternal adiponectin positively correlated with birth weight (*P* < 0.05) (Figure 1(a)). In control group maternal adiponectin negatively correlated with fetal aIMT (*P* < 0.05) (Figure 1(b)). Moreover, only IUGR and SGA maternal adiponectin negatively correlated with maternal TNF*α* (*P* < 0.05) (Figure 1(c)). In the three groups, fetal adiponectin positively correlated with maternal adiponectin (*P* < 0.05) (Figure 1(d)).

**Figure 2:**
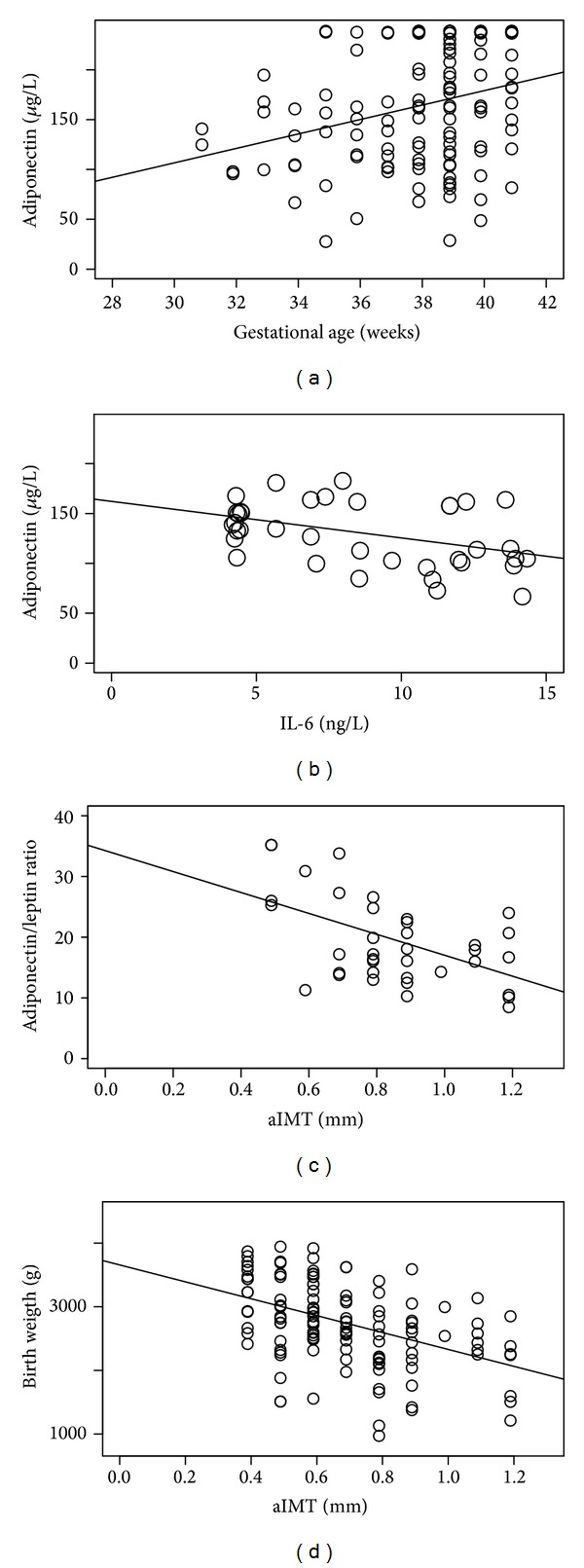
Correlations of fetal blood adiponectin, other hormone levels, anthropometric, or ultrasound parameters. (a) Correlation between gestational age at delivery and fetal blood adiponectin levels in all the population (tau test *P* < 0.05). (b) Correlation between fetal blood IL-6 levels and fetal blood adiponectin levels in IUGR population (*P* < 0.05). (c) Correlation between fetal aIMT and fetal adiponectin/leptin ratio in IUGR population (*P* < 0.05). (d) Correlation between fetal aIMT and neonatal weight in all the population (*P* < 0.05).

Furthermore, fetal adiponectin positively correlated with gestational age at delivery (*P* < 0.05) (Figure 2(a)). In IUGR fetuses, adiponectin serum concentrations were negatively correlated with fetal IL-6 (*P* < 0.05) (Figure 2(b)) and fetal adiponectin/leptin ratio (A/L) presented a negative correlation with aIMT (*P* < 0.05) (Figure 2(c)). In all groups, there was a negative correlation between aIMT and birth weight (*P* < 0.05) (Figure 2(d)).

**Figure 3:**
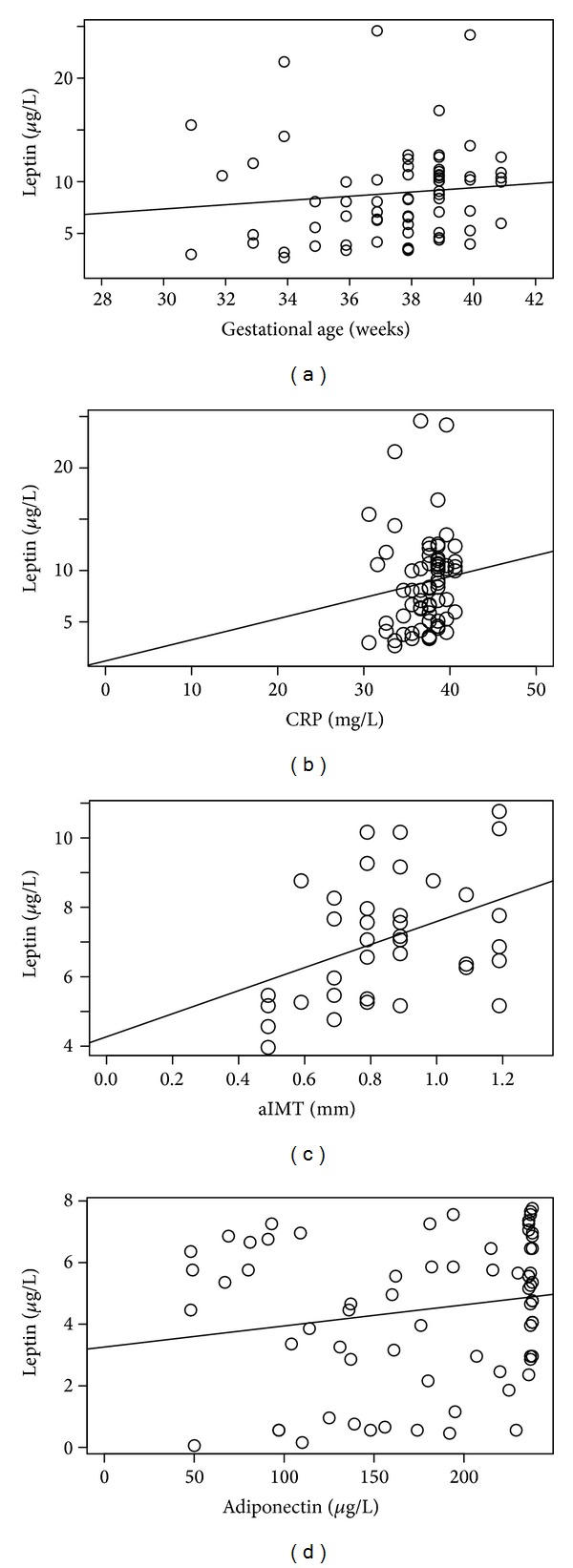
Correlations of blood leptin, other hormone levels, anthropometric, or ultrasound parameters. (a) Correlation between gestational age at delivery and maternal blood leptin levels in IUGR and SGA population (tau test *P* < 0.05). (b) Correlation between maternal blood CRP and leptin levels in IUGR and SGA population (*P* < 0.05). (c) Correlation between fetal aIMT and fetal blood leptin levels in IUGR population (*P* < 0.05). (d) Correlation between fetal blood adiponectin and leptin levels in AGA population (*P* < 0.05).

IUGR and SGA maternal serum leptin concentrations were positively correlated with gestational age at delivery (*P* < 0.05) and maternal CRP levels (*P* < 0.05) (Figures 3(a) and 3(b)).

IUGR fetal leptin levels positively correlated with fetal aIMT (*P* < 0.05) (Figure 3(c)). Only in control fetal group there was a positive correlation between adiponectin and leptin levels (*P* < 0.05) (Figure 3(d)).

## 4. Discussion

This study showed that fetuses with IUGR and Doppler abnormalities presented thicker aIM, higher concentrations of leptin, TNF*α*, IL-6, and CRP, and lower adiponectin levels than SGA or AGA. To the best of our knowledge, this is the first report demonstrating a correlation between A/L ratio and aIMT in IUGR fetuses, supposing a link between immune system and endothelial damage. This association was not found for SGA fetuses. The subdivision of IUGR disorder considering Doppler velocimetry allowed stratification into different classes of vascular risk. In SGA fetuses aIMT was lower than IUGR but higher than AGA, while adipokines and inflammatory cytokines presented only minimal differences in comparison to control group. These results confirmed previous studies in which aIMT was inversely related to EFW, showing that low birth weight and Doppler abnormalities may be correlated with an altered vascular structure causing possible endothelial damage, both in single and twin pregnancies [8, 20]. Moreover, in children who had IUGR, aIMT was greater in those with the lowest birth weight, suggesting that atherogenesis and an increased arterial stiffness may be a potential mechanism mediating the mentioned epidemiological link between impaired fetal growth and cardiovascular disease in adulthood, similar to major environmental risk factors such as cigarette smoking and hypertension [7, 21]. Postmortem studies in young adults showed an inverse correlation between birth weight and severity of aortic lesions [22]. Histochemical analysis also confirmed that the fetal aIMT observed during pregnancy by ultrasound corresponded to intima thickening. The CD68, a widely used marker for macrophages, is usually absent in normal vessels; E-selectin, a marker of activated endothelial cell (EC) and CD31, a marker for quiescent EC, were found present in the aortic wall of IUGR stillbirth. These might represent peculiar elements of preatherosclerotic lesions [23]. Experimental evidence have demonstrated that cardiovascular remodeling, triggered in response to the stress conditions in utero, persists as a permanent feature in postnatal life, including vascular dysfunction, increased blood pressure, and aorta intima media thickness [24].

In many instances, metabolic disorders as well as other disorders associated with IUGR have an endocrine origin and are accomplished by the changes in hormone bioavailability in adulthood [25]. Several independent observations have shown a relationship between low birth weight and insulin resistance; reduced insulin sensitivity might be secondary to altered programming of metabolic pathways in presence of adverse intrauterine environment [26]. IUGR fetuses showed a marked reduction in body fat mass, which mainly reflects a decreased accumulation of lipids in the adipocytes. However, although total body fat percentage is reduced, visceral adipose tissue is relatively increased and it results hyperresponsiveness to catecholamine and early insulin resistance [5]. Adipokines, bioactive molecules produced by adipose tissue, should regulate body metabolism and are implicated in fetal growth. Adiponectin influences carbohydrate metabolism, improving insulin sensitivity, and low adiponectin levels have been suggested to play a causal role in the development of insulin resistance and cardiovascular disease in adulthood [27]. In agreement with literature the present study shows that during third trimester umbilical cord blood adiponectin concentration is approximately three times higher than in maternal blood, in all groups analyzed [28]. In accordance with Lindsay, our study found that maternal adiponectin is positive with birth weight [29]. Pregnancy is a unique situation in which there is a physiological, temporary insulin resistance, gradually settled down in the third trimester, with an increase of fetal blood glucose and free fatty acid concentrations and a reduction in maternal insulinemia [30]. This could explain the reduction of maternal adiponectin at the end of pregnancy. In IUGR and SGA pregnancies, maternal adiponectin concentration negatively correlates with maternal TNF*α*, suggesting a prevalent inflammatory condition in a mother whose pregnancy is complicated by fetal growth restriction.

Adiponectin represents antiatherogenic and anti-inflammatory properties suppressing the macrophages proinflammatory cytokines production, such as TNF*α* and IL-6 [31], and inhibiting macrophage to foam cell transformation [32]. A reduction in IUGR fetuses of adiponectin level and its negative correlation with fetal IL-6 might represent the immune system's modification, which could explain the endothelial damage expressed by a thickening of aIM. Moreover, the A/L ratio, actually the most indicative sign of metabolic risk, is even negatively correlated in IUGR fetuses with aIMT [33]. Animal models reveal that the majority of macrophages in established atherosclerotic lesions are derived from local proliferation rather than from the influx of blood-borne monocytes [34]. Recent studies demonstrate the role of perivascular adipose tissue dysfunction in cardiovascular inflammation and oxidative stress [35]. Significant infiltration of macrophages and T cells in perivascular adipose tissue was accompanied by endothelial dysfunction. Decreased secretion of adiponectin and increased production of cytokines from dysfunctional adipose tissue may significantly contribute to vascular inflammation, insulin resistance, vascular stiffness, and impaired relaxation [36].

In many studies investigating the effect of low birth weight on metabolic diseases in later life, IUGR has been used equivalently to the term SGA. In contrast to SGA, IUGR implies an underlying pathological process that prevents the fetus from achieving its growth potential and can be assessed by prenatal ultrasound and Doppler examinations. In our study we found a decrease in adiponectin levels only in IUGR fetuses, and although not significant SGA presented a higher value than controls. A possible explanation for these contradictory results may be related to the different definition of IUGR, often without considering Doppler velocimetry. and in methodological aspects. Several authors described lower levels of adiponectin in SGA fetuses and children, proposing that this downregulation might be a predisposing factor for later development of insulin resistance and metabolic syndrome. Interestingly very low adiponectin levels in IUGR children should predict the subsequent development of visceral fat and insulin resistance in adulthood [37–39]. When Briana and Lindsay found similar levels of adiponectin in cord blood of SGA and control fetuses [29, 31], conversely, López-Bermejo et al. studying prepuberal children found increased adiponectin concentrations related to increased insulin sensitivity [33, 40]. Kyriakakou et al. used Doppler velocimetry in IUGR definition, finding that leptin and adiponectin serum levels were higher and lower, respectively, in IUGR fetuses, in accordance with our results [17].

Leptin seems to be a critical factor for overall fetal development. In this respect, numerous animal studies indicated that prenatal exposure to maternal under nutrition leads to the development of diet-induced obesity, hyperleptinemia, hyperinsulinism, and hypertension in the rat offspring [41]. Thus, leptin may play a role in the control of substrate utilization and in the maintenance and functional characteristics of fat mass before birth, producing permanent changes concerning adiposity and body composition in adult life [42]. In accordance with other studies, IUGR presented a positive correlation between maternal leptin and gestational age at delivery, indicating in these patients a possible preexisting metabolic alteration [40]. Moreover, in IUGR fetuses there was a positive correlation between leptin and IL-6 levels, underlying a similar proinflammatory role. The inversely correlation between fetal A/L ratio and aIMT might represents a link between endocrine function of adipose tissue and endothelial damage. In literature, there is no accordance among investigators about cord leptin concentration in this category of fetuses. Several studies demonstrated lower circulating leptin concentrations in IUGR fetuses, due to reduced fat mass and/or decreased placental production, increasing and becoming higher in IUGR infants, children, and adults [42–45], while other investigators determined similar and higher leptin concentrations [31, 46].

IUGR ovine models showed that leptin levels are inversely related to uterine blood flow and fetal/placental weight, suggesting that fetal leptin may be involved in an adaptive response [47]. Tzschoppe et al., differentiating the two groups by EFW and pathological uterine and umbilical artery Doppler velocimetry, found that leptin mRNA and protein expression are increased in the placentas of IUGR newborns compared to AGA. Hypoxic and inflammatory processes inducing placental dysfunction might explain increased placental leptin mRNA expression. Leptin gene in fact is highly sensitive to oxygen abundance and IUGR fetuses, exhibiting severe distress and having significantly higher leptin concentrations per kilogram of weight [46, 48, 49].

TNF*α* and IL-6 are produced by adipose tissue monocytes and macrophages and also by the placenta. Few and contradictory data exist in the literature regarding the IUGR state [50]. Some investigators documented a reduced fetal IL-6 and TNF*α* levels in growth restricted fetuses [51, 52], possibly due to impaired placental insufficiency. On the other hand, an upregulation of IL-6 and TNF*α* in IUGR fetuses could be secondary to hypoxia and to survival mechanism, by inducing muscle insulin resistance and enabling glucose to be spared for brain metabolism [10, 53]. In this study, we hypothesized that higher levels in IUGR fetuses could be secondary to the reduction of adiponectin concentrations, which do not inhibit macrophage-cytokines release; this condition should worsen the endothelial damage of intrauterine growth restriction. In IUGR mothers this finding might reflect the state of inflammation and chronic stress, expressed also by high levels of CRP, not found among IUGR, SGA, and AGA fetuses. High sensitivity CRP was not measured, and this might explain our result.

In conclusion, a specific profile of increased leptin, IL-6, CRP, and TNF*α* in IUGR mothers might indicate a proinflammatory condition for the development of poor intrauterine environment. The increased umbilical leptin, TNF*α*, and IL-6 concentrations and the decreased adiponectin levels in IUGR fetuses might represent the inflammatory substrate that contributes to the vessel remodelling, represented by thickening of the aorta. These conditions could predispose to vascular and metabolic disorders in adult life. Differential regulation of adipocytokines and higher aIMT in utero in the IUGR state may be predictive of adult disease. Further understanding of the changes in adipocyte maturation during prenatal nutrition and their influence on molecular pathways could help explain the complex association between IUGR and adult disease risk and support the development of effective preventive strategies.

## Figures and Tables

**Table 1:** Description of the samples. Data are expressed as mean ± standard deviation or percentage. The *P* value refers to *t*-test, chi-square test, or Fisher's exact test.

	IUGR (37)	SGA (33)	Controls (70)	*P*
Woman age (years)	32.70 (±4.4)	31.90 (±5.1)	32.00 (±4.9)	NS
Prepregnancy BMI (kg/m^2^)	23.80 (±6.3)	20.80 (±2.9)	22.10 (±2.9)	(1, 3)
Nulliparous women	69%	55%	53%	NS
Mode of delivery				
Spontaneous delivery	39%	33%	54%	NS
Caesarean section	61%	67%	46%	NS
Fetal gender				
Male	42%	27%	54%	(3)
Gestational age at delivery (weeks)	36.75 (±2.82)	38.45 (±1.59)	38.57 (±2.14)	(1, 2)
Neonatal weight (grams)	2131.67 (±519.4)	2648.23 (±282.8)	3178.17 (±510.7)	(1, 2, 3)
aIMT (mm)	1.10 (±0.20)	0.80 (±0.20)	0.60 (±0.20)	(1, 2, 3)

Significant differences (*P* < 0.05) between (1) IUGR and SGA; (2) IUGR and AGA; and (3) SGA and AGA.

NS: nonsignificant differences.

BMI: body mass index.

aIMT: aorta intima media thickness.

**Table 2:** Differences in examinations of blood values among the evaluated groups in maternal and fetal circulation. Data are presented as median and interquartile range (IQR). The *P* value refers to Wilcoxon test.

	IUGR (37)	SGA (33)	Controls (70)	*P*
Maternal blood				
Adiponectin (*µ*g/L)	45 (32–61)	58 (37–64)	57 (45–61)	(2)
Leptin (*µ*g/L)	8.2 (5–11.9)	8.5 (5.2–10.7)	5.5 (3.1–7.6)	(2, 3)
Adiponectin/leptin	5.5 (2.7–8.7)	5.9 (4.6–12.5)	11.4 (7.1–18.1)	(2, 3)
IL-6 (ng/L)	4.9 (2.3–8.4)	3.9 (2.5–5.4)	4.6 (2.4–6.2)	NS
TNF*α* (ng/L)	5.4 (4.3–10)	5.0 (4–5.6)	4.9 (4.0–6.1)	(1, 2)
CRP (mg/L)	5.1 (3.9–12.8)	4.1 (2.8–5.7)	4.9 (2.9–5.9)	(1, 2)
Fetal blood				
Adiponectin (*µ*g/L)	134 (105–159)	197 (122–239)	196 (134–239)	(1, 2)
Leptin (*µ*g/L)	7.1 (5.5–8.3)	4.3 (2.7–7.8)	4.9 (2.9–6.5)	(1, 2)
Adiponectin/leptin	17.4 (14.3–24.2)	43.5 (24.4–75)	41.3 (31.1–79.7)	(1, 2)
IL-6 (ng/L)	8.6 (4.6–12.1)	5.3 (2.4–8.9)	4.7 (2.3–6)	(1, 2, 3)
TNF*α* (ng/L)	11.1 (8.9–14.9)	8.3 (7.5–9.1)	8.7 (7.3–9.9)	(1, 2)
CRP (mg/L)	2.8 (2.7–2.9)	2.8 (2.7–2.9)	2.8 (2.8-2.9)	NS

Significant differences (*P* < 0.05) between (1) IUGR and SGA; (2) IUGR and AGA; and (3) SGA and AGA.

NS: nonsignificant differences.
